# Effects of Enzymatically Depolymerized Low Molecular Weight Heparins on CCl_4_-Induced Liver Fibrosis

**DOI:** 10.3389/fphar.2017.00514

**Published:** 2017-08-21

**Authors:** Yishu Yan, Changge Guan, Shanshan Du, Wenming Zhu, Yang Ji, Nan Su, Xiang Mei, Dong He, Yuan Lu, Chong Zhang, Xin-Hui Xing

**Affiliations:** ^1^MOE Key Laboratory of Industrial Biocatalysis, Institute of Biochemical Engineering, Department of Chemical Engineering, Tsinghua University Beijing, China; ^2^Bio-Cell Co., Ltd. Beijing, China; ^3^Centre for Synthetic and Systems Biology, Tsinghua University Beijing, China

**Keywords:** LMWHs, non-anticoagulant activity, liver fibrosis, comparative pharmacology, heparinase, heparin depolymerization

## Abstract

With regard to identifying the effective components of LMWH drugs curing hepatic fibrosis disease, we carried out a comparative study on the efficacy of enzymatically depolymerized LMWHs on CCl_4_ induced mouse liver fibrosis. The results showed that the controlled enzymatic depolymerization conditions resulted in LMWHs with significantly different activities. The LMWH product depolymerized by Heparinase I (I-11) with a Mw of 7160, exhibited a significant advantage in reducing the liver inflammation by suppressing TNF-α and IL-1β secretion, and minimizing hepatic fibrogenesis. The products prepared by only Heparinase II (II-11), and combined Heparinase III and II (III-II-5) showed limited positive effect on hepatic inflammation and fibrosis. On the contrary, the products by combined Heparinase III and I (III-I-9, III-I-5) showed no effect or stimulation effect on the hepatic fibrogenesis. Our results provided the basis for structure-activity relationship insight for inhibition of liver fibrosis activities of LMWHs, which might have significant implications for generic anti-fibrosis disease drug development.

## Introduction

Tissue fibrosis represents a conserved pathological process, during which iterative injury in any organ triggers excess extra cellular matrix deposition (Rockey et al., 2015). Liver fibrosis is a key step to cirrhosis, liver failure, portal hypertension, and a high risk of developing hepatocellular carcinoma. This pathological process contributed to around 1.5 million deaths per year (Hernandez-Gea and Friedman, 2011), and become a significant health problem worldwide (Fagone et al., 2016). Currently only Pirfenidone and Nintedanib have been approved as anti-fibrosis drugs, however, not specific for liver fibrosis. The clinical available drugs all acted against virus or metabolic syndrome (Poelstra, 2016). The effective pharmacotherapy targeting liver fibrosis is thus extremely urgent.

Heparin and low molecular weight heparins (LMWHs) are a group of linear glycosaminoglycans. Variable patterns of substitution of the disaccharide subunits with sulfate groups give rise to a large number of complex sequences (Rabenstein, 2002). They are classical anti-coagulant agents in clinic (Lever and Page, 2002). Their non-anticoagulant activities have been explored widely (Page, 2013). However, the discovery and development of non-anticoagulant heparin is currently limited by the undetermined structure and activity relationship. Indeed, each therapeutic activity of heparin has its specific structural requirement, and the presence of sequences without relation to the targeting activity may add risk of side effects (Mousavi et al., 2015). On the other hand, the heterogeneous and complicated nature of heparin chains made it difficult to separate, purify and characterize the sequences with high selectivity (Jones et al., 2011).

Based on the existing data, it also seemed encouraging to treat hepatic fibrosis with heparin and LMWHs. They have been used to alleviate portal vein thrombosis (PVT) of cirrhosis, delay the occurrence of hepatic decompensation and improve survival of patients in clinical trials (Villa et al., 2012; Qi et al., 2014). At the same time, there were increasing findings about the beneficial effect beyond the anti-coagulant activities (Liu et al., 2009; Cerini et al., 2016). They decreased the hepatic fibrosis by suppression of the transforming growth factor-β (TGF-β)/Smad pathway, up-regulation of hepatocyte growth factor, and inhibition of hepatic stellate cell (HSC) proliferation by the PDGF augmented both expression and phosphorylation of extracellular signal-regulated kinase proteins and activator protein-1 activity (Li et al., 2006; Abe et al., 2007; Lee et al., 2011). Yet the anti-fibrotic mechanisms are distinct according to different manufacturing processes. It’s not possible to screen for the effective components against single target *in vitro*.

Contradictory results for curing liver fibrosis also exist. Administration of Tinzaparin, a main species of commercial available LMWHs, increased fibrosis area compared to negative control group (Abdel-Salam et al., 2005). The reasons behind this contradiction may be attributed to the different depolymerization processes resulting in distinct structural entities, which account for considerable differences of pharmaceutical activities (Yan et al., 2017b).

For discovery of the candidates responsible for anti-liver fibrosis activity, we herein constructed a depolymerized LMWHs library by an individual or combined heparinases (Hep I, II, or III). We compared the performance of LMWHs using CCl_4_ induced hepatic fibrosis model, and identified the components that showed the activities in slowing the disease progression. Our results provided the basis for further structure-activity relationship insight for inhibition of liver fibrosis activities of LMWHs.

## Materials and Methods

### Materials

Carbon tetrachloride (CCl_4_) was obtained from Beijing Chemical Factory (China). Aspartate aminotransferase (AST), and alanine aminotransferase (ALT) determination kits were purchased from Nanjing Jiancheng Bioengineering Institute (China). Pharmaceutical grade heparin (Mn = 22,370, anti-factor Xa activity = 187 ± 21 IU/mg, anti-factor IIa activity = 177 ± 6 IU/mg) was purchased from the Changshan Biotechnology Corporation (Hebei Province, China). All the reagents, solvents and chemicals were of analytical grade. The primary antibody used in the present study was against α-smooth muscle actin (α-SMA) (Sigma-Aldrich). ELISA kits for interleukin-1β (IL-1β), interleukin-6 (IL-6) and tumor necrosis factor α (TNF-α) were purchased from Proteintech Group Inc., United Kingdom. The Catalog Numbers were KE10002, KE10003, and KE10007, respectively. PrimeScript^TM^ RT-PCR Kit was purchased from Takara Bio (Otsu, Japan). SYBR Green PCR Master Mix was purchased from Takara Bio (Otsu, Japan). Sirius Red kits were purchased from Sigma–Aldrich (the United States).

### Animals

C57BL/6 mice weighing 20–25 g were purchased from the Laboratory Animal Research Center of Tsinghua University (Beijing, China). In compliance with the *Guide for Laboratory Animal Care and Use*, all the animals received humane care and had free access to food and water during the study. All the animal procedures were approved by the Laboratory Animal Care and Use Committees of the Tsinghua University.

### Methods

#### Production and General Characterization of Enzymatically Depolymerized Heparins

MBP-Heparinases (MBP-Hep), including MBP-Hep I, MBP-Hep II, and MBP-Hep III, were a group of enzymes with distinct specificities, which were used to depolymerize heparin into various LMWHs motifs. In our previous study, a controllable production process of LMWHs by a combinational use of heparinase I/II/III has been set up (Wu et al., 2014). By using this strategy, a series of LMWHs were generated for examining their anti-liver fibrotic activities.

The depolymerization degree of heparin by individual MBP-Hep I/II/III was controlled by measuring absorbance at 232 nm. For example, the preparation process for Hep I depolymerized product (I-11) can be described as followed: 1 mL of Hep I solution with an enzyme activity of 15 IU/mL was added to a 100 mL reaction solution (pH = 7.4) containing 50 g/L heparin, 20 mM Tris, 200 mM NaCl, and 5 mM CaCl_2_. The enzymatic reaction was monitored at 232 nm with an ultraviolet spectrophotometer (Gold spectrum lab 54, Lingguang Tech., China). When A232 was detected to be 46.3, the reaction was stopped by putting into boiling water bath for 5 min. For the depolymerization of heparin by the combination of MBP-Hep III/I or MBP-Hep III/II, heparin was first exhaustively depolymerized by Hep III overnight. Then, the resulting products were further depolymerized by Hep I and II, respectively. The depolymerized products were obtained as a precipitate after six times volume addition of ethanol, and then dried by lyophilization for the subsequent analysis.

The measurements of the molecular weight (Mw, Mn), polydispersity index (P) of the depolymerized products, and the determination of anti-coagulation (anti-factor Xa and anti-factor IIa) activities were performed according to European Pharmacopoeia. More specially, gel permeation chromatography was used to analyze the size distributions (**Table 1**). Their anti-coagulant activities were listed in **Table 2**.

**Table 1:** The depolymerized heparins and their molecular weight distribution.

Products (Number^∗^)	Reaction end point A (232 nm)		Molecular weight and distribution				
		Mw/Da	Mn/Da	<3 K (%)	3–5 K (%)	5–8 K (%)	>8 K (%)
I-11	46.3	7160	3768	22.273	15.842	21.674	40.211
II-11	46.5	10255	4073	4.524	8.110	16.860	70.506
III-I-5	59	4007	1595	57.292	20.037	14.573	8.098
III-I-9	111.2	2809	1219	79.341	13.961	5.401	1.297
III-II-5	61	7122	3298	24.988	19.681	26.001	29.330
III-II-9	84	6077	2779	31.681	22.304	24.794	21.221

*^∗^The products were numbered as the Enzyme-Number of the product. For example, I-11 represents the 11th product produced by Hep-I; III-II-5 represents the 5th product produced by combined Hep- III and Hep-II.*

**Table 2:** The anticoagulant activity (anti-factor Xa activity/anti-factor IIa activity) of depolymerized heparins.

Product number	Anti-F-Xa (IU/mg)	Anti-II-a (IU/mg)
I-11	89.95–98.15	41.3–45.7
II-11	89.9–98.1	92.5–97.8
III-I-9	55.1–57.4	20.0–20.7
III-I-5	91.1–93.2	55.6–59.1
III-II-5	105.6–112.3	110.0–114.5
III-II-9	81.0–82.1	66.3–72.9

#### CCl_4_-Induced Liver Injury and Fibrosis

Eight-week-old male C57BL6 mice were randomly divided into eight groups (*n* = 8). The drug testing groups were injected with CCl_4_ dissolved in olive oil (40:60) twice a week. The dose was 4 mg/kg each time for the first 4 weeks, and 2.5 mg/kg for the next 4 weeks (i.p.) (Sakaida et al., 2004). Positive control (PC) group was set up by giving only the same volume of olive oil without CCl_4_.

Different groups of the enzymatically depolymerized heparins, or the same volume of saline as negative control (NC) group, were injected to the mice (i.p.) after 12 h since CCl_4_ injection. All of the animals were sacrificed by cervical dislocation under anesthesia after 8 weeks. The blood was collected from heart immediately before euthanasia. The tissues of spleen, and liver were removed for the assays described below (Satoh et al., 2017).

#### Hematoxylin-Eosin (H&E) Staining, Sirius Red Staining and Immunohistochemical Analysis (Fischer et al., 2008)

At the end of experimental period (8 weeks), the mice were sacrificed, and liver tissues were immediately taken out. Most of the tissues were transferred to ice-cold containers containing 0.9% NaCl for the subsequent various biochemical examinations.

The intermediate lobes were fixed with 4% paraformaldehyde solution in PBS. The paraffin embedded liver tissue sections (5 μm in thickness) were stained with H&E, and Sirius Red according to standard techniques. Histological analyses were referred to the literature. To see the expression level of liver fibrosis markers, the liver sections were stained with antibodies against α-SMA at a dilution under standard immunohistochemical procedures.

#### Hydroxyproline (Hyp) Determination

Approximately 100 mg wet liver tissue for each mouse was placed in 60°C incubator for 24 h to remove the water inside. The Hyp levels in the liver tissue (100 mg) were determined according to Hyp detection kit (Nanjing Jiancheng Bioengineering Institute, China). The Hyp content was expressed in micrograms of Hyp per milligram of dry weight (μg/mg) (Satoh et al., 2017).

#### Determination of Enzymatic Activity in Mouse Serum

The serum was collected before the mice were sacrificed, and ALT, AST levels in the serum were analyzed on ultraviolet visible light spectrophotometer (Gold spectrum lab 54, Lingguang Tech., China). The procedures were according to the assay kits.

#### Enzyme-Linked Immunosorbent Assay (ELISA)

The antibodies used for ELISA were included in the colorimetric sandwich ELISA kit. The monoclonal antibodies specific for TNF-α, IL-1β, and IL-6 were pre-coated onto the microwells.

Liver tissue was homogenized with 10-fold saline, followed by centrifugation. The resulting supernatant was then diluted by 10-fold for quantitative assay of the inflammatory factor expression levels. The resulting 100 μL samples were added to each well of the 96-well plates, followed by incubation for 2 h at room temperature. Working detector solution of 100 μL was loaded into each well, and the plates were incubated for an additional 1 h at room temperature before the addition of 100 μL substrate solution. The reaction was stopped by adding 50 μL of stop solution. The absorbance was read at 450 nm wavelength. Values were normalized to control.

#### Real-Time PCR

Total RNA from the whole livers of the mice was reversely transcribed into cDNA (Applied Biosystems). The transcription level of the fibrosis relevant genes was analyzed by quantitative real-time PCR on an ABI 7300 system (Advanced Biosystems, Foster, CA, the United States) using SYBR Green PCR Master Mix. Each measurement was repeated in triplicate and normalized to the corresponding GAPDH content values. Details can be found in the literature (Chen et al., 2013).

#### Statistical Analysis

Results were means ± SD for biological replicates. Differences between two groups were calculated by independent-samples *t*-test. Significance of differences between groups was determined by one-way ANOVA.

## Results

### Enzymatically Depolymerized LMWHs Exhibited Different Effects on Physiological Parameters of Mice with CCl_4_ Induced Hepatic Fibrosis

**FIGURE 1:**
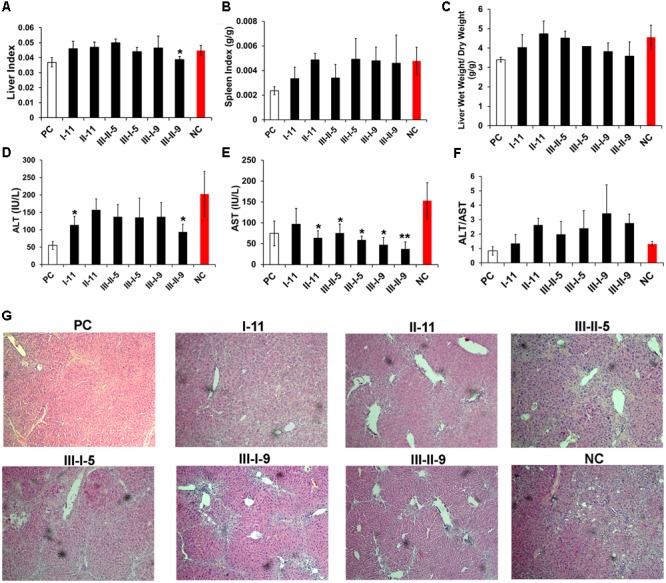
Primary efficacy screening with the enzymatically depolymerized heparins on mice with CCl_4_-induced liver fibrosis. Liver indexes **(A)**, spleen indexes **(B)**, wet/dry weight of liver **(C)**, the activity of serum ALT **(D)**, AST **(E)**, and ALT/AST **(F)** were tested to determine the hepatic functions. **(G)** The morphological changes of hepatic tissues were examined by H&E staining. Results are means ± SD for biological replicates (*n* = 4, 5). ^∗^ was taken as minimum level of statistically significant differences of LMWHs groups against the NC group (*P* < 0.05); ^∗∗^ was taken as minimum level of statistically very significant differences of LMWHs groups against the NC group (*P* < 0.01).

After continued administration of CCl_4_ for 8 weeks, the survival percentage for NC, I-11 and III-I-5 group was 50% (4 of 8 mice survived), whereas it was 62.5% for II-11, III-I-9, III-II-5 and III-II-9 groups (5 of 8 mice survived). Then, the survived mice were sacrificed soon after the LMWHs injection. The physiological parameters were initially evaluated for estimating the liver functions. First, the results showed that chronic CCl_4_ administration alone induced a pronounced increase in both spleen index (wet spleen weight/body weight) and liver index (wet liver weight/body weight) compared with PC group. However, the LMWHs exerted different performances on the two pathological indicators. Only III-II-9 group decreased the liver index, whereas other LMWHs appeared to have no impact (**Figure 1A**); I-11, and III-II-5 treatment significantly counteracted the increase of the spleen index, whereas other LMWH groups showed no reduction compared with the NC group (**Figure 1B**). The liver edema was considered with regard to wet weight/dry weight, which increased rapidly in response to CCl_4_ induced liver injury in NC group. Our results revealed that I-11, III-I-5, III-I-9, and III-II-9 all decreased the ratios, indicating all the tested LMWHs alleviated the edema (**Figure 1C**).

The serum transaminases (ALT, AST, and ALT/AST) were further evaluated for assessment of the hepatocellular damage of the model mice (**Figures 1D–F**). In the controlled liver fibrosis model, a dramatic increase in the liver enzymatic activities was observed, whereas all the LMWHs groups suppressed the ALT and AST levels, suggesting that LMWHs could ameliorate liver injury induced by CCl_4_ administration. However, only I-11 group reduced the ratio of ALT/AST to a normalized level, which is an important reflection of the liver function; on the contrary, other groups of the LMWHs increased the ratio of ALT/AST.

The pathological histology of the livers was evaluated by H&E staining (**Figure 1G**). The CCl_4_ induction resulted in severely pathological changes in the liver, including disordered hepatocyte, serious steatosis, and inflammatory cells infiltration. The pseudolobule, and bridging bands might indicate collagen deposition along the blood vessels. Treatment with I-11 and II-11 resulted in attenuation of tissue damage with alleviated hepatocytes degeneration and collagen deposition than that of the NC group. The III-I-9, III-II-9, III-II-5, and III-I-5 groups, however, didn’t show obvious attenuation effect. Taken together, the preliminary results have shown that I-11 group treatment exhibited the best performance on attenuating CCl_4_-induced liver inflammation in mice.

### LMWHs Regulated CCl_4_ Induced Hepatic Injury and Inflammation

**FIGURE 2:**
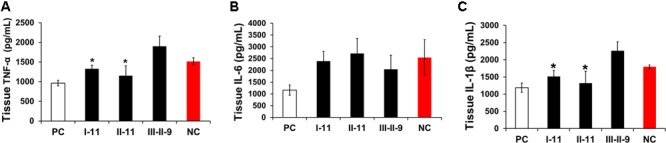
Effect of enzymatic depolymerized LMWHs on liver inflammation of mice with hepatic fibrosis. The inflammation activities of livers were evaluated by TNF-α **(A)**, IL-6 **(B)**, IL-1β **(C)** expression levels in liver homogenate. Results are means ± SD for biological replicates (*n* = 4, 5). ^∗^ was taken as minimum level of statistically significant differences of LMWHs groups against the NC group (*P* < 0.05).

Inflammation commonly lead to liver fibrosis, and is associated with the fibrosis process (Seki and Schwabe, 2015). To further explore the underlying mechanisms of I-11 protection of the liver from CCl_4_ induced injury, we evaluated the expression of pro-inflammatory mediators, TNF-α (**Figure 2A**), IL-6 (**Figure 2B**), and IL-1β (**Figure 2C**) of hepatic homogenate by ELISA assays, with II-11 and III-II-9 group as comparison. The results demonstrated that these mediators all elevated significantly in fibrotic liver in NC group. I-11 and II-11 groups showed the same trend in regulating the inflammation by abolishing the TNF-α, IL-1β elevations, but didn’t affect the IL-6 expression levels; whereas III-II-9 group has shown the opposite trend by stimulating TNF-α and IL-1β secretion, and slightly suppressed the expression level of IL-6. As I-11 showed the strongest attenuation effect, we supposed I-11 suppressed inflammation by selective downregulation of TNF-α and IL-1β.

### Comparison of Anti-fibrotic Effects of Enzymatically Depolymerized LMWHs on Mice with CCl_4_ Induced Hepatic Fibrosis

**FIGURE 3:**
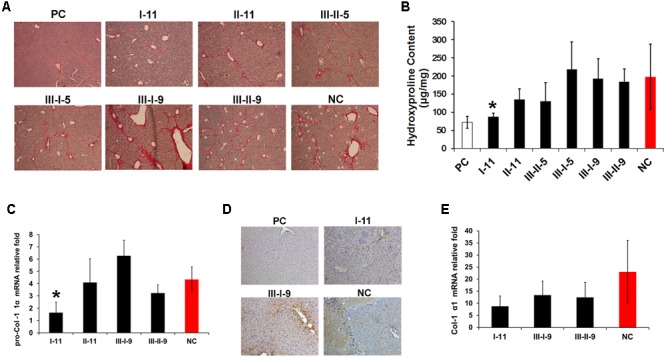
Anti-fibrotic effect evaluation of different enzymatic depolymerized products on of CCl_4_ induced mouse hepatic fibrosis model. **(A)** Comparison of effect on collagen deposition with enzymatic depolymerized LMWHs by Sirius Red staining. **(B)** Analysis of whole liver collagen content by Hyp assay. **(C)** Real-time PCR analyses of pro-Col I α1 expression level. Results are means ± SD for biological replicates (*n* = 4, 5). ^∗^ was taken as minimum level of statistically significant differences of LMWHs groups against the NC group (*P* < 0.05); ^∗∗^ was taken as minimum level of statistically very significant differences of LMWHs groups against the NC group (*P* < 0.01). **(D)** Liver sections were immunohistochemical stained with α-SMA antibody. **(E)** Real-time PCR analyses of Col I α1 expression level.

Liver fibrosis in mice after administration of CCl_4_ was first confirmed by Sirius Red staining (**Figure 3A**). The results revealed that Sirius Red staining was confined to the portal tracts in the livers of PC group. Collagen staining was observed in the area of stretches from portal area to lobular in the NC group; meanwhile, septa were noticed, and additional cirrhotic nodule formation was visualized. I-11 group had minimum collagen deposition, II-11 and III-II-5 groups also exhibited less collagen, but no significant differences between III-II-9, III-I-5 and NC groups were observed. On the opposite, III-I-9 group showed a trend of collagen deposition increasement.

To quantitatively evaluate the fibrosis deposition, liver Hyp content was measured (**Figure 3B**). The results showed a significant elevated level in NC group, and decreased most in response to I-11 group. Whereas II-11, III-II-5 group also showed different degree of reduced Hyp accumulation in comparison with the NC group. The results consisted with the above data, indicating that I-11 showed significant effect in slowing the progression of liver fibrosis.

To further prove the efficacy of I-11, accumulated collagen was characterized by measuring the mRNA expression of pro-Collagen I α1 (**Figure 3C**) and Collagen I (**Figure 3E**), with III-I-9 and III-II-9 groups as comparison. The expression level of the two markers exhibited a great enhancement in the NC group compared with the PC group. Consistent with the previous results, I-11 treatment abolished the expression greatly. Concurrently, III-I-9 group promoted the mRNA expression of pro-Collagen I α1, and suppressed Collagen I α1 expression slightly; III-II-9 treatment didn’t show significant change compared with the NC group. Moreover, the enhanced expression of α-SMA, a definite marker for fibrosis disease, was observed in liver tissue in NC group (**Figure 3D**) by immunohistochemical staining. A substantial reduction of α-SMA expression was observed in response to I-11 treatment. Taken together, these results confirmed that I-11 administration could cause reduced accumulation of extracellular matrix that resulted in the liver fibrosis and cirrhosis.

## Discussion

In this paper, we carried out a comparative study of effects with the enzymatically depolymerized LMWHs by single or combination of Hep I, II, and III on CCl_4_ induced mouse liver fibrosis. According to the results of Sirius Red staining and Hyp determination, we could draw the conclusion that I-11 exhibited the best anti-fibrotic activities by reducing the deposition of collagen; II-11, III-II-5 showed limited effect on fibrosis reduction compared with I-11 group. But no differences between III-II-9, III-I-5 and NC groups were observed. On the opposite, III-I-9 group showed increased collagen deposition by Sirius Red staining. Besides, we have confirmed the anti-fibrotic effect of I-11 group by determination mRNA’s expressions of α-SMA, pro-Collagen I α1, Collagen I α1 etc. These data clearly supported that I-11 has shown the best activity in inhibition of hepatic injury and fibrosis.

The activity differences among species of LMWHs could be attributed to the distinctive structures by different depolymerization conditions. It’s believed specific heparin structure be responsible for their anti-liver fibrosis activities. Heparinases have different selectivities. Hep I depolymerizes heparin on GlcNS(±6S)-IdoA(2S); whereas Hep III acts on a very rare sequence of GlcNS/Ac(±6S)-GlcA/IdoA. Therefore, the resulting sequences of products by different heparinases are distinct (Wu et al., 2014). The effective anti-fibrotic components might be destroyed during the III-II-9, III-I-5 group production, whereas Hep I protect the effective components from depolymerization during I-11 production.

However, structural heterogeneity of LMWHs makes the structure-activity relationship analysis challenging. The structure and anti-fibrosis activity relationship has not been determined yet. In the present study, we have proved I-11, the depolymerized product of Hep I exhibited an attractive application potential for treatment of liver fibrosis; while treatment by combined Hep III/I depolymerized products showed the increment of liver fibrosis. Further comparison of the structure of the depolymerized products will provide the critical structure information responsible for anti-liver fibrosis activity.

The fact I-11 inhibited liver fibrosis did not mean Hep I protect the effective components from depolymerization. For example, Tinzaparin, a species of commercial available Hep I depolymerized LMWHs, increased fibrosis area with BDL models. Compared with Tinzaparin, I-11 has higher percentage in high molecular weight distribution (40.211 vs. 22–36%, >8 K), but lower percentage in medium molecular weight distribution (45.896 vs. 60–72%, 2 K < Mw < 8 K). Therefore, we suppose the component with larger molecular weight might be responsible for the fibrogenesis activity.

Indeed, we found that LMWHs with larger molecular weight had better fibrosis inhibition activity compared with that of the smaller one. For example, III-II-5 (with a Mw of 7122) showed limited liver fibrogenesis inhibition activity, while III-II-9 group (with a Mw of 6077) showed no effect. This leads to an impetus for fine controlled process for producing LMWHs with narrow molecular weight distribution, which in turn may provide the basis for studying the specific LMWH sequences contributing to the anti-fibrotic role.

Activation of the coagulation cascade is one of the earliest events initiated after tissue injury, after which inflammation, fibroproliferation, and remodeling sequentially occurred. Therefore, coagulation has been regarded as a pivotal role in tissue repair process (Calvaruso et al., 2008). In the present study, we measured the anticoagulant activities of different depolymerized heparins (**Table 2**). However, the results didn’t demonstrate the trends the products with high anticoagulant activity prevent hepatic fibrosis progress.

TNF-α and IL-1β, in particular, have been identified as the important targets in a variety of fibrotic diseases (Wynn and Ramalingam, 2012). Activation of HSCs was regarded as a key event in the initiation of liver fibrogenesis. In the microenvironment of liver fibrosis, secreted IL-1β and TNF-α have been shown to promote the survival of activated HSCs and increase the inflammation reaction (Pradere et al., 2013). Moreover, they have been shown to promote the survival of HSC-derived myofibroblasts, and the development of liver fibrogenesis via an MMP/TIMP imbalance (Pellicoro et al., 2014; Robert et al., 2016).

Herein we have proved that I-11 affected hepatic fibrosis disease by selectively downregulation of IL-1β and TNF-α. We suppose the next step research on I-11’s effect on the fibrosis cascade should direct at the regulatory pathways of TNF-α and IL-1β, respectively. However, LMWHs have been proved to be highly pleiotropic drugs as anti-inflammatory agents, suppressing the excess expression of various pro-inflammatory mediators (Saito and Tabata, 2012; Li et al., 2015). In addition, fibrosis deposition, inflammation and uncontrolled coagulation systems crosstalked during the fibrosis diseases (Calvaruso et al., 2008). We need further research to determine whether the antifibrotic effects of I-11 are attributable to one pathway or a combination of several.

More broadly, fibrosis is a highly conserved and universal pathological process, suggesting common signal pathways and therapeutic targets (Seki and Schwabe, 2015). Therefore, the findings in the treatment of liver fibrosis could be possibly extended to other organs. Indeed, heparin prevented/slowed down the disease process including cystic fibrosis (Serisier et al., 2006), peritoneal fibrosis (Li et al., 2015), and idiopathic pulmonary fibrosis (Gunther et al., 2003). In the concurrent study, we have optimized the structure for anti-pulmonary fibrosis treatment (Yan et al., 2017a). Theoretically speaking, this collaborative multi-organ approach, combined with the detailed structural comparison of effective components, could accelerate the drug development of novel generic LMWHs for curing fibro-proliferative diseases in the future.

## Author Contributions

YY designed, finished most of the animal test, and drafted the manuscript. WZ, SD and CG did the animal test, analyzed the data. YJ and NS did all the ELISA experiments. XM, DH, YL, and CZ put forward with the conception. X-HX drafted and modified the manuscript for important intellectual content.

## Conflict of Interest Statement

The authors declare that the research was conducted in the absence of any commercial or financial relationships that could be construed as a potential conflict of interest.
